# Cardiac-Specific Cre Induces Age-Dependent Dilated Cardiomyopathy (DCM) in Mice

**DOI:** 10.3390/molecules24061189

**Published:** 2019-03-26

**Authors:** Taha Rehmani, Maysoon Salih, Balwant S. Tuana

**Affiliations:** Department of Cellular and Molecular Medicine, Faculty of Medicine, University of Ottawa, Ottawa, ON K1H 8M5, Canada; trehmani@uottawa.ca (T.R.); msalih@uottawa.ca (M.S.)

**Keywords:** cardiac-specific-cre, dilated cardiomyopathy, cre-lox system, mouse model, cardiotoxic, DNA damage

## Abstract

The genetic modification of the mouse genome using the cre-lox system has been an invaluable tool in deciphering gene and protein function in a temporal and/or spatial manner. However, it has its pitfalls, as researchers have shown that the unregulated expression of cre recombinase can cause DNA damage, the consequences of which can be very detrimental to mouse health. Previously published literature on the most utilized cardiac-specific cre, αMHC-cre, mouse model exhibited a nonlethal hypertrophic cardiomyopathy (HCM) with aging. However, using the same αMHC-cre mice, we observed a cardiac pathology, resulting in complete lethality by 11 months of age. Echocardiography and histology revealed that the αMHC-cre mice were displaying symptoms of dilated cardiomyopathy (DCM) by seven months of age, which ultimately led to their demise in the absence of any HCM at any age. Molecular analysis showed that this phenotype was associated with the DNA damage response through the downregulation of activated p38 and increased expression of JNK, p53, and Bax, known inducers of myocyte death resulting in fibrosis. Our data urges strong caution when interpreting the phenotypic impact of gene responses using αMHC-cre mice, since a lethal DCM was induced by the cre driver in an age-dependent manner in this commonly utilized model system.

## 1. Introduction

Genetic manipulations in model organisms are the crux of biological and medical research in terms of ascribing physiological or pathological roles for the gene of interest [1]. Through modifications of a model organism’s genome, it has been possible to discover, verify, and establish complex roles of genes and the proteins they encode [2]. The genetically modified mouse model is very attractive, as mice share a very similar genetic profile, anatomy, and disease progression to humans [3]. Genetic modifications of the mouse take place through the manipulation of mouse embryonic stem cells by taking advantage of the stem cells’ natural inclination toward homologous recombination to selectively replace the endogenous target gene sequence with modified exogenous DNA [4]. Significant strides in genetic engineering have allowed us to utilize many models and systems for modifying the genome, such as the cre-lox system [5].

The cre-lox system is a site-specific recombination system used to modify DNA through manipulation derived from the P1 bacteriophage [6]. It is a two-part system where the recombinase, cre, binds to a specific 34-base pair sequence known as a loxP site [7]. To employ cre-lox, the gene of interest is modified to be flanked by two loxP sites (floxed), and thus when the cre enzyme is introduced it cleaves the flanked sequence and merges the two excision points together, resulting in a smaller sequence and thus permanently modifying the floxed gene [8,9]. The use of cre-lox is enticing because the modification by the cre recombinase of the gene target, can be controlled in a temporal and spatial manner [10]. Specific promoter sequences spliced with cre recombinase result in tissue-specific expression, excising floxed genes only within the specific cell of interest [11]. The fusing of *cre* to modified estrogen receptors (Mer) retains cre in the cytosol until it is activated by an estrogen analog, tamoxifen [12]. The simplicity of cre, with no additional requirements for recombination (such as cofactors or accessory proteins) and loxP sites which are not endogenous in mammals, makes the cre-lox system an ideal candidate for genetic engineering in cells and animal models [13].

To target the floxed genes of mouse myocardium, researchers have implemented the use of the cardiac specific promoter α-myosin heavy chain (*αMHC*) to ensure that cre is expressed only within cardiomyocytes [14]. This specific cre has been widely utilized for targeting floxed genes within the mouse heart to elucidate gene function and impacts on the myocardium [15,16]. This system, however, has demonstrated pitfalls [15,16,17].

Overactive cre recombinase can be toxic, as studies have shown that it can inhibit DNA replication and increase DNA damage: However, regulating the concentration or activity of cre can minimize toxicity [18]. This may be due to the presence of endogenous lox-like sequences within the mammalian genome that can be targeted by cre [19,20]. Evidence suggests that cre is cardiotoxic, as numerous publications using the inducible *αMHC-MerCreMer* have shown that large doses of tamoxifen overstimulate cre, resulting in decreased cardiac function through enhanced myocyte death/fibrosis and DNA damage [21,22]. The focus has been mostly on the MerCreMer model: However, in a non-inducible αMHC-cre model, there has only been one study highlighting the development of a nonfatal hypertrophic cardiomyopathy (HCM) that presents in six-month-old mice [16]. We report here that in the same αMHC-cre mice, an age-dependent fatal dilated cardiomyopathy (DCM) was prevalent. We believe this to be an important finding, as this cardiac-specific cre model has been used in over 150 studies, with 14 publications highlighting an age-dependent fatal DCM attributed to their respective gene modifications without utilizing the appropriate cre-only controls in a timed manner. The DCM appeared to be a byproduct of cre cardiotoxicity, resulting from an accumulation of DNA damage. We hope our results will further highlight the pitfall of using long-term cardiac-specific cre as well as encourage the use of proper cre-only controls during gene manipulation studies in the postnatal heart.

## 2. Results

### 2.1. The Change in Lifespan of αMHC-cre Mice

**Figure 1:**
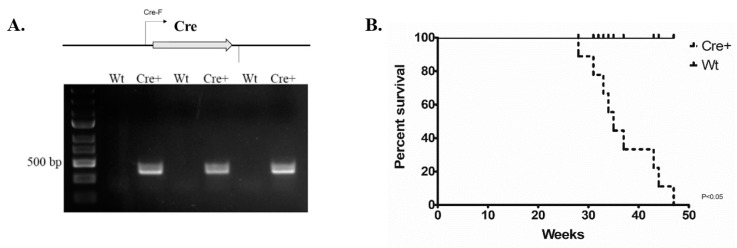
Long-term exposure to αMHC-cre resulted in complete lethality in mice by 11 months of age. (**A**) PCR genotyping using primers Cre-F/Cre-R to identify Wt or Cre+ littermates. Wt lanes indicate a wild-type genotype, whereas Cre+ lanes indicate the presence of *αMHC-cre* (425 bp). (**B**) Kaplan–Meier survival curve analysis of Cre+ mice compared to Wt littermates. Cre+ animals began dying at 7 months, dropping off intermittently until 11 months of age (*p* < 0.05, *n* = 11).

We employed mice that express αMHC-cre that have been used in a large variety of cardiac gene manipulation studies [14]. The genotype of these animals was determined through PCR to differentiate wild-type (Wt) littermates and establish the αMHC-cre only (Cre+) experimental animals. Primers *Cre-F* and *Cre-R* were used to detect *cre* by synthesizing a DNA amplicon 425 base pair (bp) in length: Wt animals lack such an insert (seen in respective agarose gel lanes (Figure 1A)). We examined 11 Cre+ and 11 wild-type littermates to determine any impact of timed cre exposure inthe myocardium and on mouse survival (Figure 1B). We observed a 50% loss of Cre+ mice by approximately 8 months of age, with the remainder dying before 11 months of age (Figure 1B**)**. All Cre+ animals were seen to be in pain and distress (body condition scoring = 2−) [23]. Thus, mice carrying *αMHC-cre* underwent age-dependent death, with complete loss within one year of age. This data is consistent with the notion that cre is cardiotoxic and that this particular cre mouse model exhibits timed lethality [15].

### 2.2. αMHC-cre Mice Presented with an Age-Dependent Dilated Cardiomyopathy

**Figure 2:**
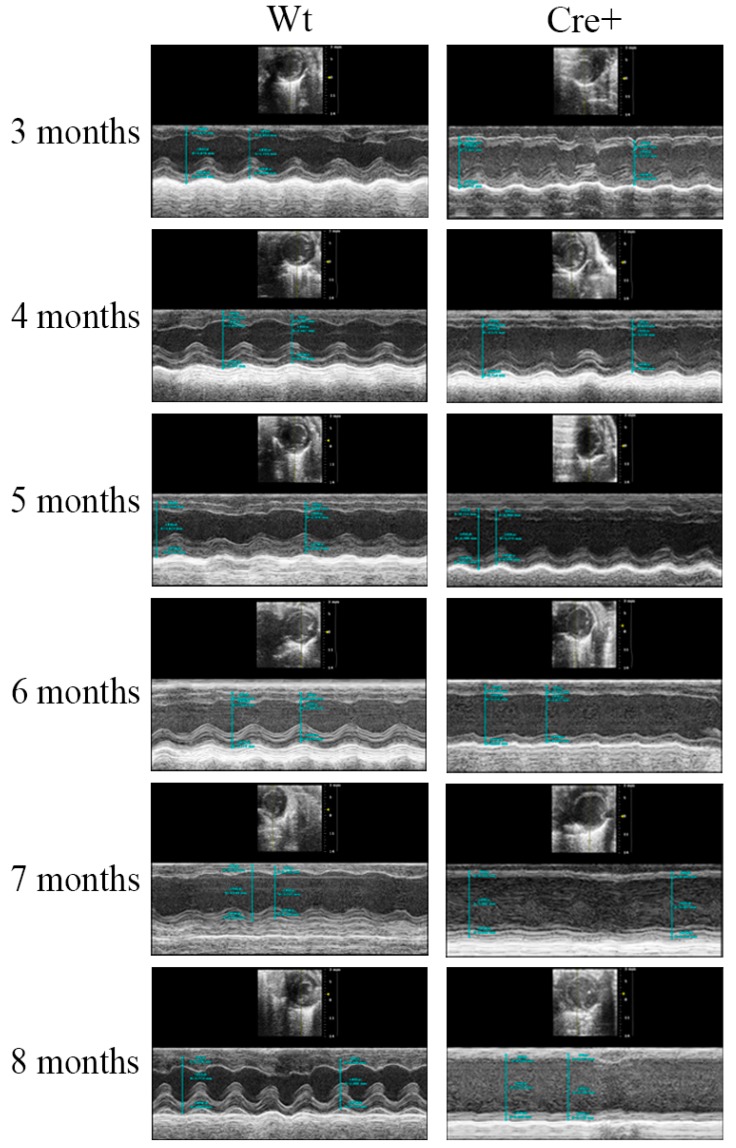
Echocardiography imaging revealed significant comparisons between aging Wt and Cre+ hearts. Representative short-axis m-mode transthoracic echocardiography (ECHO) images of wild-type and Cre+ animals at 3–8 months of age (*n* = 8).

**Figure 3:**
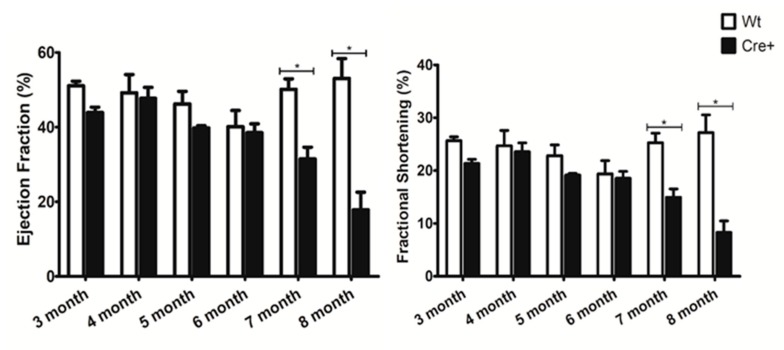
Age-dependent changes in cardiac function to Wt and Cre+ hearts. Cardiac function was analyzed with ECHO and the calculated ejection fraction and fractional shortening in Wt and Cre+ animals 3–8 months of age is presented. (* indicates a *p* value less than 0.05, *n* = 8).

Given the age-dependent lethality noted in the αMHC-cre mice, we examined any changes in cardiac structure and function to discover the cause. Transthoracic echocardiography (ECHO) was performed in an age-dependent manner in mice, beginning at three months of age and repeated monthly until they perished. The left ventricle wall thickness (interventricular septum (IVS), left ventricle posterior wall (LVPW)) and the left ventricle internal diameter (LVID) in systole and diastole were determined. These measurements were used to evaluate cardiac function by calculating the ejection fraction (EF), fractional shortening (FS), which measure percentage of blood output and muscle contraction respectively [24]. Table 1 shows a comprehensive evaluation of the data acquired at different time points for ECHOs in Wt and Cre+ mice. Before six months of age, αMHC-cre and Wt mice had identical left ventricle structure and function (Figure 2). However, at six months of age, the left ventricle wall of αMHC-cre was thinner with the presence of dilation compared to Wt hearts (Figure 2). At seven months, a significantly thinner IVS;d (18.62% decrease, *n* = 8, *p* < 0.05) and LVPW;d (16.3% decrease, *n* = 8, *p* < 0.05) and a significantly larger ventricle interdiameter at systole (29.5% increase, *n* = 8, *p* < 0.05) in Cre+ hearts compared to Wt was notable (Table 1). An analysis of these changes indicated a significant reduction in the EF (37.25% decrease, *n* = 8, *p* < 0.05) and FS (40.76% decrease, *n* = 8, *p* < 0.05) in Cre+ hearts when compared to Wt (Figure 3). Thinning of the left ventricle wall and a dilation of the chamber were clear indications of a lethal phenotype: Dilated cardiomyopathy (DCM). This phenotype was further exacerbated as αMHC-cre animals aged, resulting in an even greater change in cardiac structure and significantly diminished function at eight months of age (Figure 2 and Figure 3). Therefore, αMHC-cre induced an age-dependent DCM, which significantly reduced cardiac function and resulted in observable respiratory distress and death.

### 2.3. Lack of Hypertrophy and Robust DCM in αMHC-cre Mice

**Figure 4:**
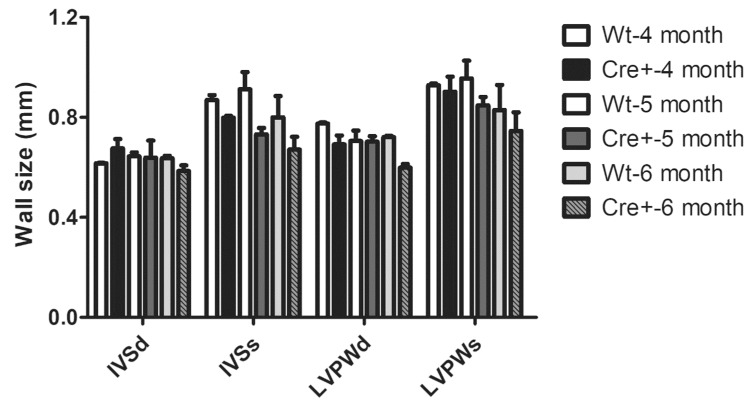
Left ventricular wall parameters in female Wt and Cre+ hearts. Left ventricle wall thickness of 4 to 6-month-old female mice. Here, *n* = 3. IVS: Interventricular septum; LVID: Left ventricular internal diameter; LVPW: Left ventricular posterior wall; d/s: Diastole/systole.

Here, αMHC-cre mice exhibited a notable age-dependent DCM that led to their demise. A previous study indicated the presence of a hypertrophic cardiomyopathy at 6 months of age in these αMHC-cre mice [16]. To determine if the αMHC-cre mice developed any HCM, we evaluated the wall thickness of mice beginning at 3 months of age, and no observable or significant thickening of ventricle walls was seen in ECHOs from the Wt or Cre+ animals (Table 1). Sex-separated analysis on 4–6-month-old female Cre+ mice versus Wt littermates indicated no difference in hypertrophy (Figure 4). In fact, female Cre+ hearts at 6 months of age trended toward ventricular wall thinning, not thickening (Figure 4). Thus, the collective (male/female) or female-only ECHO data suggested that αMHC-cre exhibited a robust DCM in an age-dependent manner in the absence of any notable HCM.

### 2.4. αMHC-cre Mice Exhibited Dilated Hearts and Fibrosis

**Figure 5:**
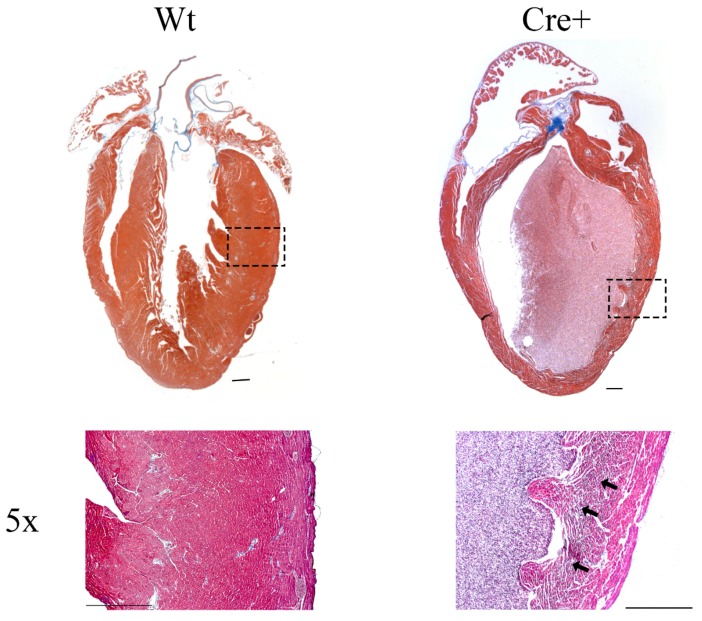
Histological analysis of 8-month-old Wt and Cre+ hearts. Representative images of 8-month-old wild-type and Cre+ mouse hearts that underwent histological sectioning and staining, imaged under a brightfield microscope. Masson’s trichrome stain was used to visualize the myocardium walls (red) and fibrosis (blue). In addition, 5× magnification using a brightfield microscope was used to identify the presence of fibrosis in Wt or Cre+ hearts, marked with black arrows in fibrotic regions. Scale bar = 500 µm.

The presence of a lethal DMC in the aged αMHC-cre mice was surprising and was further examined by histological analysis. Hearts from 8-month-old wild-type and αMHC-cre mice were fixed, sectioned, and stained with Masson’s trichrome to visualize the myocardium (red stain) and acquire evidence of any overt fibrosis (blue stain). Hearts from the Cre+ mice had significantly thinner walls and a dilated chamber compared to Wt (Figure 5). Magnification of the image at 5× revealed significant blue staining indicative of fibrosis in the Cre+ hearts (arrows). The thin walls, dilated ventricle, and evident fibrosis confirmed that aged αMHC-cre mice were exhibiting DCM, and this was noticeably absent in the hearts of Wt mice [25].

### 2.5. A DNA Damage Response Presented in αMHC-cre Myocardium

**Figure 6:**
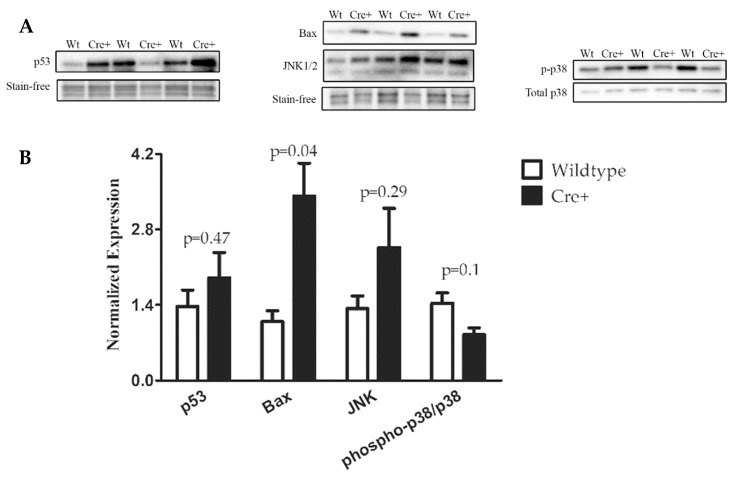
Expression of DNA damage markers in Wt and Cre+ hearts. (**A**) Western blot was utilized to determine the expression of DNA damage related (p53, p38, JNK) to the pro-death protein Bax in 8-month-old Wt or Cre+ hearts. (**B**) Bar graphs representing relative expressions of p53, Bax, and JNK normalized to total proteins visualized by stain-free technology (Bio-Rad, Hercules, CA, USA). Phospho-p38 was normalized to total p38.

**Figure 7:**
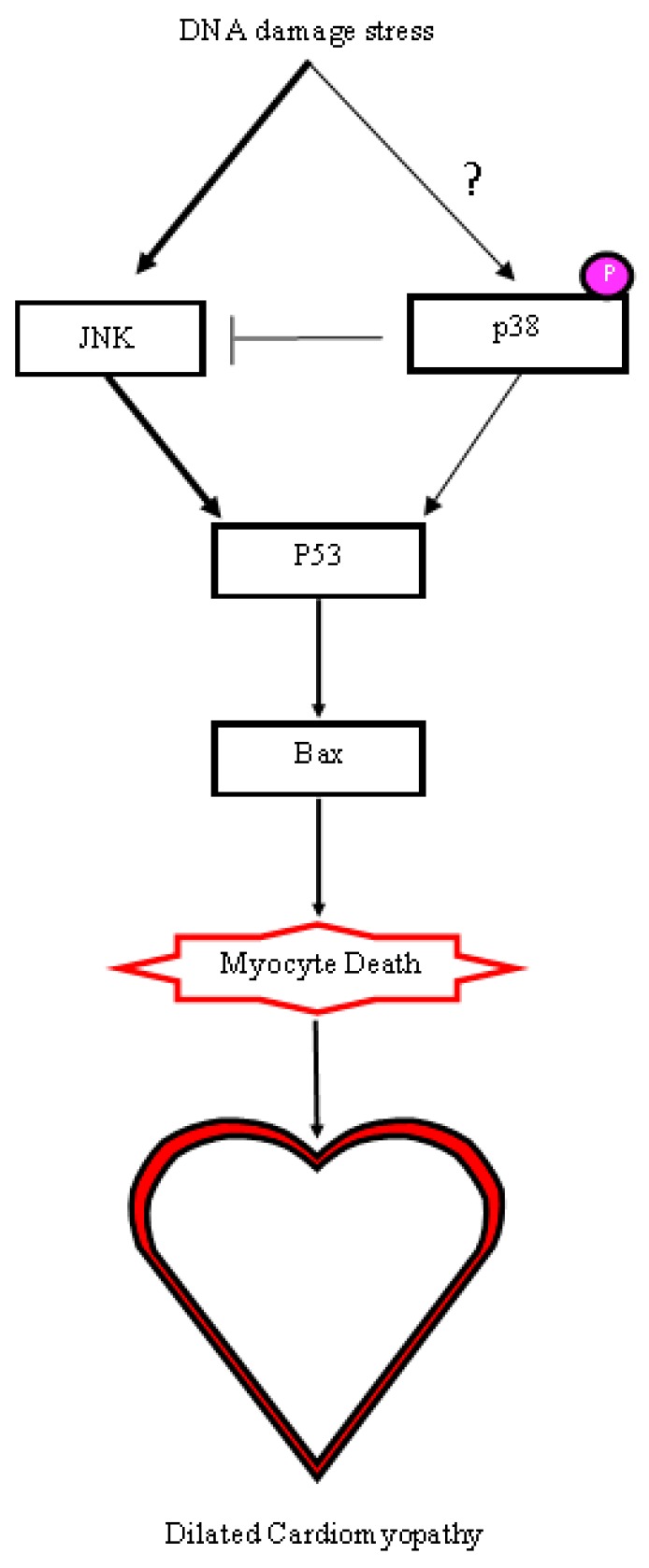
Proposed molecular mechanism for the induction of DCM in αMHC-cre mice. The decrease in p38 activity enhanced JNK, resulting in an upregulation of the p53/Bax causing myocyte death. The change in p38 activity due to cre-induced DNA damage and external signaling initiated a cascade that eventually resulted in DCM.

Cre recombinase has been shown to cause DNA damage when overly stimulated or highly expressed [26]. Though steps are taken to reduce the amount of cre in cells, hemizygous (one allele) MerCreMer animals have been shown to cause phenotypes when overly stimulated by tamoxifen [21,22]. The αMHC-cre mice were examined for any changes in DNA damage by evaluating phospho-p38 and p53, both shown to be upregulated when DNA stress is applied [27,28]. The relative amount of DNA stress response proteins assessed with western blots of lysates isolated from 8-month-old Wt and αMHC-cre myocardium (Figure 6) showed a sizable increase in the expression of p53 in two Cre+ hearts, while no change was noted in one of the Cre+ hearts compared to Wt hearts (38.9% ± 41.5%, *n* = 3, *p* = 0.47) . A substantial decrease of phosphorylated p38 in two Cre+ hearts was observed, while the other remained unaffected when compared to Wt (−40.8% ± −16.8%, *n* = 3, *p* = 0.10). JNK (c-Jun NH_2_ Kinases) is negatively regulated by p38, while JNK itself has been demonstrated to positively regulate p53 [29,30]. Western blot data indicated that all three Cre+ hearts had a modest increase in JNK (84.9% ± 59.3%, *n* = 3, *p* = 0.29) compared to Wt (Figure 6). The death protein Bax, which is a direct downstream target of p53 signaling, was significantly increased in Cre+ myocardium (213.8% ± 71.4%, *n* = 3, *p* = 0.04) compared to Wt myocardium (Figure 6) [31]. Taken together, the expression data showed that αMHC-cre resulted in increased amounts of DNA damage, which activated the stress kinase pathway, potentially causing myocyte loss and fibrosis, leading to DCM (Figure 7).

## 3. Discussion

The cre-lox system is an important tool that is used to determine gene function in many different models. The toxicity of cre is well documented, and without the use of proper controls, it can go unobserved. In this study, we showed that the most utilized αMHC-cre mice could develop an age-dependent DCM in the absence of hypertrophy. This cardiac phenotype is unique and distinct from recently published data using the same αMHC-cre mice, where the mice developed an age-dependent nonlethal HCM [16]. Histological analysis via Masson’s trichrome staining of 8-month-old hearts clearly identified thin walls, a dilated chamber, and fibrosis in the Cre+ hearts, which are typical symptoms of DCM. ECHO analysis showed that by seven months of age, the left ventricle walls of Cre+ hearts were significantly thinner and dilated compared to wild-type hearts. Interestingly, no observable hypertrophy was present in αMHC-cre hearts at any age examined or when sex-separated analysis was performed. The accumulation of DNA damage, as seen with the increase in p53 and the pro-death protein Bax (which are associated with apoptosis and cell death), was notable in αMHC-cre hearts [32,33]. p53 has been demonstrated to be impacted by DNA damage and directly targets the expression of genes involved in cell cycle arrest and apoptosis. [27]. Phosphorylation of p38 has been shown to increase in the presence of environmental stress and signal the launch of an apoptotic response [29,34]. Our data indicated a markedly decreased activation of p38 and an increase in JNK in Cre+ hearts, providing an alternative mechanism of myocyte death, as p38 has been shown to negatively regulate JNK [35,36]. The upregulation of JNK positively regulated the activity of p53, potentially leading to Bax-mediated myocyte death and the increased fibrosis notable in the dilated αMHC-cre myocardium, as modeled in Figure 7 [37,38,39]. There was considerable variation in the level of DNA damage within the αMHC-cre hearts, although they all exhibited a robust DCM. While individual (two) αMHC-cre hearts exhibited some very dramatic changes, no overall significant changes to p53, JNK, and p38 were evident due to variation (one heart) among the cohort. This indicated that there were multiple factors that impacted the exhibition of the DNA damage response in αMHC-cre myocardium, which in turn may determine the extent of the cardiac phenotype.

Cardiomyopathy is associated with structural changes within the myocardium due to pathological remodeling, when the heart enlarges the left ventricle to compensate for a pressure or volume overload [40,41]. The mechanism through which αMHC-cre causes cardiomyopathy remains unclear, as two phenotypes (HCM or DCM) were found to be expressed within the same cardiac-specific cre model and mouse strain. The molecular changes to p53 and Bax were similar, with the cell death pathways activated leading to the two different phenotypes i.e., DCM or HCM. However, it is difficult to reconcile how myocyte cell loss would lead to an HCM given that robust fibrosis was apparent [16]. The one difference noted between the two studies, however, was the upregulated expression of phoshpo-p38 seen in HCM [16]. Studies have demonstrated that activated p38 signals a hypertrophic response in cardiomyocytes through the upregulated gene expression of atrial natriuretic peptide, brain natriuretic peptide, and alpha-skeletal actin [42]. Further, the consistent activation of p38 displayed dilated cardiomyopathy in adult mouse hearts probably due to depressed function as a consequence of the fetal gene program [43]. However, a decreased activation of p38 was found in patients exhibiting symptoms of DCM and end-stage heart failure [44], a molecular signature and phenotype similar to the aged cre mice in our studies. Our data showed that the decreased activity of p38 could impact JNK and transgenic mice with increased JNK activity exhibited DCM, while overactive JNK was reported in patients exhibiting idiopathic DCM [38,45,46,47]). p38 and JNK are stress kinases regulated by extracellular signals, which can be influenced through differences in animal husbandry, diets, genetics, and environmental stresses [34]. DCM or HCM has also been attributed to such diverse factors [48,49]. Although the specific effect of the molecular changes in p38 and JNK in the mouse heart remain controversial, there is a definite impact on cardiac health [47,50]. Therefore, molecular changes due to DNA damage (and its extent) induced by cre exposure are likely impacted by diverse factors, including environmental stress, which in turn may determine distinct pathology.

DCM has been previously implicated in two different cardiac-specific cre models, indicating the extremely cardiotoxic nature of the cre recombinase. The use of tamoxifen stimulates the activation of αMHC-MerCreMer, resulting in a transient DCM that may result in lethality with a tamoxifen overdose [21] while the high-level expression of αMHC-cre resulted in mice perishing before reaching 3 months of age [51]. This αMHC-cre mouse model was a different strain, FVB, compared to the C57B6 strain utilized here. The FVB model was generated under different conditions, which yielded offspring with different expressers of cre recombinase, with high expressers leading to DCM in young mice and lower expressers not showing any phenotypes although the impact of age was not examined [51]. Thus, regulating the amount of cre is critical in hearts, and the use of low-expressing cre can minimize off-target effects. However, consistent with other reports [15,16,51] we further stressthat caution, even with the low-level expression of αMHC-cre, as two cardiac phenotypes, due to chronic cre exposure, can be exhibited in an age-dependent fashion. Fourteen studies that have utilized αMHC-cre mice have reported age-dependent DCM, which was attributed to a respective genetic modification. However, cre-only controls at the appropriate age were lacking or not shown [52,53,54,55,56,57,58,59,60,61,62,63,64,65]. Further, in many of the studies lacking cre controls, the respective gene targets (EP4, ESRRβ, ATE1, Mdm4, NEMO/IKKgamma, the glucocorticoid receptor) had not been previously or subsequently shown to cause DCM [52,53,54,55,56,57]. Our data strongly urges the use of an appropriate cre control in a timed manner which will reveal a more accurate result of any impact of the genetic modification of interest.

In conclusion, our findings show that caution must be exercised when performing long-term studies using αMHC-cre mice, as different cardiac phenotypes can arise from low-level chronic cre exposure. The use of cre-only controls is critical and interrogating the impact of environment on the expression of DCM versus HCM in these αMHC-cre mice may help reveal the mechanisms differentiating the two phenotypes.

## 4. Materials and Methods

### 4.1. Maintenance of αMHC-cre Mouse Colonies

The αMHC-cre mice were generously donated by Dr. Patrick Burgon (University of Ottawa, Faculty of Medicine, Ottawa) for our experiments. The mouse colony was maintained through backcrossing transgenic males with wild-type C57B6 females (Charles Rivers Laboratories, Wilmington, MA, USA). Mice were genotyped by extracting genomic DNA from ear clips by boiling for 10 min at 95 °C in 180 μL of 50 mM NaOH per ear. DNA solution was then neutralized using 20 μL of 1 M Tris-Cl, pH 8.0. The αMHC-cre (Cre+) or wild-type (Wt) mice were identified by polymerase chain reaction (PCR) using a DreamTaq Green PCR Master Mix 2× (Thermo Fisher Scientific, Waltham, MA, USA) for the PCR mix. To determine Cre+ animals, we utilized forward primer Cre-F (5′-ACG ACC AAG TGA CAG CAA TG-3′) and reverse primer Cre-R (5′-AAC CAG CGT TTT CGT TC-3′). PCRs were visualized by Red Safe (Sigma-Aldrich, St. Louis, MO, USA) staining on a 1% agarose gel.

Mice were handled in accordance with the guidelines set by Canadian Council on Animal Care, Guide to the Care and Use of Experimental Animals, 2 vols. (Ottawa, Ont.: CCAC, 1980-1993) and Animals for Research Act, R.S.O. 1990, c.A. 22. All animal protocols and procedures were approved by the Animal Care Committee of the University of Ottawa.

### 4.2. Protein Isolation from Mouse Hearts

Hearts of adult mice were collected after CO_2_ euthanasia and immediately frozen at −80 °C. Each heart was later washed with ice-cold 1× phosphate buffered saline (PBS) and homogenized using a Fisher handheld Maximizer homogenizer (Thermo Fisher Scientific, Waltham, MA, USA) in ice-cold lysis buffer (1 mM ethylene glycol tetraacetic acid (EGTA), 1 mM ethylenediaminetetraacetic (EDTA), 20 mM Tris base, 1% Triton, 150 mM sodium chloride, 1× complete mini EDTA-free protease inhibitor cocktail (Roche, Basel, Switzerland), and 1× PhosSTOP (Roche, Basel, Switzerland). The suspension was centrifuged for 15 min at 12,000× *g* to separate the proteins from the cell debris. The supernatant containing protein was collected in Eppendorf tubes and stored in a freezer at −80 °C.

### 4.3. SDS-PAGE and Western Blots

For western blotting experiments, 10 µg of protein was loaded in each well of a 5–15% SDS-PAGE gel. The gels were transferred overnight on a polyvinylidene fluoride (PVDF) membrane (Bio-Rad, Hercules, CA, USA) in a buffer containing 25 mM Tris, 190 mM Glycine, and 20% methanol. All membranes were blocked at room temperature for 1 h in Tris-buffered saline (TBST) containing 1 M Tris, 290 mM NaCl, 0.1% Tween 20, pH 7.4, and 5% nonfat dry milk. Primary antibodies were incubated overnight at 4 °C with 5% bovine serum albumin (BSA). A comprehensive list of primary antibodies, manufacturers, and working dilutions are shown in Table 2. Membranes were washed 5 times for 5 min each in TBST prior to adding the appropriate horseradish peroxidase-labeled secondary antibody (Jackson ImmunoResearch, West Grove, PA, USA) in a 1:10,000 dilution in TBST with 5% nonfat dry milk. Membranes were shaken slowly at room temperature for 1 h while incubating with secondary antibody, followed by 5 washes for 5 min each with TBST. Membranes were treated with a BioRad western blotting kit (Bio-Rad, Hercules, CA, USA) and developed using ChemiDoc machines (Bio-Rad, Hercules, CA, USA). Bands were quantified by densitometry using Image Lab software v.6.0.0 (Bio-Rad, Hercules, CA, USA) Membranes were stripped (25 mM glycine, 10% SDS, and pH 2.2 in dH_2_O) and reprobed with different antibodies. When using stain-free technology, stain-free gels (Bio-Rad, Hercules, CA, USA) and low-fluorescence PVDF membranes (Bio-Rad, Hercules, CA, USA) were used.

### 4.4. Echocardiography

All echocardiographic analysis was done using the VEVO 2100 system (FUJIFILM VisualSonics, Toronto, Canada). Adult mice were anesthetized using 2% isoflurane and strapped onto a heated pad facing upwards, exposing the thoracic cavity. A 40 MHz probe was used to capture short-axis B-mode and M-mode images of the left ventricle. VEVO v1.6 software (FUJIFILM VisualSonics, Toronto, Canada) was utilized for measuring LV wall thickness and inner diameters in diastole and systole. The formulas used by the VEVO v1.6 software to calculate EF, FS, LV mass, and LV Vol;d/s are listed below:

EF: 100 × ((LV Vol;d − LV Vol;s) / LV Vol;d)(1)

FS: 100 × ((LVID;d − LVID;s) / LVID;d)(2)

LV mass: 0.8 × 1.053 × ((LVID;d + LVPW;d + IVS;d)^3^ − LVID;d^3^) (3)

LV vol; d/s: ((7.0 / (2.4 + LVID;d/s)) × LVID;d/s^3^ (4)

### 4.5. Histological Analysis

Hearts were extracted from animals and fixed using 10% Neutral Buffered Formalin (Thermo Fisher Scientific, Waltham, MA, USA). After fixing for 48 h, hearts were sectioned 4 µm longitudinally per section. Sectioned hearts were stained with Masson’s trichrome to visualize the myocardium and fibrosis.

### 4.6. Statistical Analysis

The statistical analysis was performed using GraphPad Prism software version 5 for Windows (GraphPad Software, La Jolla, SD, USA). All comparisons between wild-type and Cre+ groups were analyzed using two-tailed Student’s *T*-test. All error bars presented in graphs are represented using the standard error of the mean. All sample size values (*n*) represent biological replicates (Kaplan-Meier, westerns, and ECHO).

## Figures and Tables

**Table 1:** Left ventricular wall thickness and functional analysis of Wt and Cre+ animals. Left ventricle analysis on Wt and Cre+ mice from ECHO studies from 3 to 8 months of age. Functional analysis was done via measurements of left ventricle wall sizes and interdiameters during diastole and systole.

**3 months**	**Wt**	**Cre+**	***p*-value**	**4 months**	**Wt**	**Cre+**	***p*-value**
IVS;d (mm)	0.630 ± 0.025	0.620 ± 0.027	0.291692478	IVS;d (mm)	0.645 ± 0.028	0.675 ± 0.033	0.788462538
IVS;s (mm)	0.841 ± 0.024	0.752 ± 0.042	0.088168344	IVS;s (mm)	0.840 ± 0.031	0.799 ± 0.006	0.063367682
LVID;d (mm)	3.988 ± 0.120	3.891 ± 0.062	0.844773361	LVID;d (mm)	3.945 ± 0.130	3.816 ± 0.170	0.854738647
LVID;s (mm)	2.965 ± 0.104	3.062 ± 0.078	0.533414051	LVID;s (mm)	2.983 ± 0.210	2.923 ± 0.180	0.816015832
LVPW;d (mm)	0.752 ± 0.039	0.703 ± 0.009	0.421564695	LVPW;d (mm)	0.741 ± 0.033	0.692 ± 0.031	0.163169522
LVPW;s (mm)	0.950 ± 0.058	0.865 ± 0.023	0.186036258	LVPW;s (mm)	0.917 ± 0.021	0.903 ± 0.052	0.612779363
EF (%)	51.078 ± 1.225	43.930 ± 1.415	0.050885727	EF (%)	49.190 ± 4.900	47.703 ± 2.932	0.626142978
FS (%)	25.670 ± 0.730	21.351 ± 0.789	0.040507965	FS (%)	24.692 ± 2.920	23.568 ± 1.676	0.592817029
LV Mass (mg)	77.478 ± 6.617	69.760 ± 0.624	0.462915868	LV Mass (mg)	76.123 ± 4.930	70.596 ± 4.086	0.474495439
LV Vol;d (µL)	69.792 ± 4.975	65.625 ± 2.444	0.828743538	LV Vol;d (µL)	68.114 ± 5.262	63.086 ± 6.373	0.875915463
LV Vol;s (µL)	34.230 ± 2.917	36.890 ± 2.231	0.540609259	LV Vol;s (µL)	35.345 ± 5.924	33.372 ± 4.70	0.832144194
Heart Rate (bpm)	475.063 ± 31.085	426.813 ± 25.030	0.24795013	Heart Rate (bpm)	442.375 ± 15.083	488.813 ± 18.431	0.131686783
**5 months**	**Wt**	**Cre+**	***p*-value**	**6 months**	**Wt**	**Cre+**	***p*-value**
IVS;d (mm)	0.703 ± 0.055	0.621 ± 0.033	0.221718131	IVS;d (mm)	0.645 ± 0.0270	0.623 ± 0.025	0.582227989
IVS;s (mm)	0.867 ± 0.037	0.760 ± 0.032	0.066845485	IVS;s (mm)	0.770 ± 0.046	0.773 ± 0.036	0.944541382
LVID;d (mm)	4.015 ± 0.070	4.24 ± 0.022	0.011199612	LVID;d (mm)	3.860 ± 0.086	4.267 ± 0.050	0.000984789
LVID;s (mm)	3.010 ± 0.094	3.428 ± 0.029	0.007459428	LVID;s (mm)	3.111 ± 0.115	3.480 ± 0.073	0.015556863
LVPW;d (mm)	0.771 ± 0.045	0.720 ± 0.019	0.292980414	LVPW;d (mm)	0.741 ± 0.013	0.670 ± 0.022	0.037445862
LVPW;s (mm)	0.941 ± 0.033	0.853 ± 0.036	0.123190118	LVPW;s (mm)	0.881 ± 0.058	0.840 ± 0.038	0.533692627
EF (%)	46.214 ± 3.352	39.801 ± 0.561	0.070796387	EF (%)	40.113 ± 4.339	38.503 ± 2.380	0.728792586
FS (%)	22.836 ± 2.019	19.160 ± 0.307	0.081406368	FS (%)	19.377 ± 2.510	18.545 ± 1.313	0.75158218
LV Mass (mg)	85.180 ± 7.413	82.324 ± 3.134	0.711666602	LV Mass (mg)	73.251 ± 2.367	79.583 ± 3.594	0.228762289
LV Vol;d (µL)	70.721 ± 2.901	80.372 ± 0.981	0.010393045	LV Vol;d (µL)	64.510 ± 3.442	81.820 ± 2.206	0.000959293
LV Vol;s (µL)	38.018 ± 2.791	48.402 ± 0.994	0.006305021	LV Vol;s (µL)	38.590 ± 3.453	50.370 ± 2.421	0.015009289
Heart Rate (bpm)	468.229 ± 19.224	437.563 ± 22.691	0.470433394	Heart Rate (bpm)	456.333 ± 23.479	457.083 ± 21.667	0.985755943
**7 months**	**Wt**	**Cre+**	***p*-value**	**8 months**	**Wt**	**Cre+**	***p*-value**
IVS;d (mm)	0.652 ± 0.046	0.530 ± 0.017	0.004871736	IVS;d (mm)	0.672 ± 0.038	0.525 ± 0.020	0.002876701
IVS;s (mm)	0.852 ± 0.075	0.655 ± 0.028	0.004820388	IVS;s (mm)	0.897 ± 0.038	0.583 ± 0.023	2.01935E-05
LVID;d (mm)	4.153 ± 0.107	4.710 ± 0.085	0.00075479	LVID;d (mm)	3.992 ± 0.087	5.0120 ± 0.733	0.002527764
LVID;s (mm)	3.100 ± 0.078	4.015 ± 0.146	0.000380906	LVID;s (mm)	2.911 ± 0.181	4.613 ± 0.218	0.000506287
LVPW;d (mm)	0.731 ± 0.035	0.612 ± 0.0260	0.010122283	LVPW;d (mm)	0.729 ± 0.043	0.607 ± 0.029	0.037276373
LVPW;s (mm)	0.960 ± 0.063	0.735 ± 0.033	0.001783791	LVPW;s (mm)	0.899 ± 0.048	0.658 ± 0.047	0.009337212
EF (%)	50.140 ± 2.792	31.463 ± 3.171	0.000971708	EF (%)	53.049 ± 5.311	17.836 ± 4.746	0.001064173
FS (%)	25.264 ± 1.830	14.966 ± 1.571	0.000618349	FS (%)	27.203 ± 3.350	8.287 ± 2.190	0.000655345
LV Mass (mg)	83.423 ± 9.092	80.60 ± 2.389	0.667096037	LV Mass (mg)	78.378 ± 3.758	90.433 ± 6.474	0.242979928
LV Vol;d (µL)	76.765 ± 4.640	103.311 ± 4.489	0.001168324	LV Vol;d (µL)	69.821 ± 3.655	120.755 ± 9.005	0.00328618
LV Vol;s (µL)	38.055 ± 2.364	71.912 ± 6.740	0.002004722	LV Vol;s (µL)	33.135 ± 5.253	100.210 ± 10.261	0.001357712
Heart Rate (bpm)	458.750 ± 33.700	488.292 ± 20.507	0.509364526	Heart Rate (bpm)	426.833 ± 45.470	503.890 ± 12.685	0.308473653

IVS: Interventricular septum; LVID: Left ventricular interdiameter; LVPW: Left ventricular posterior wall; EF: Ejection fraction; FS: Fractional shortening; LV mass: Left ventricular mass; LV volume: Left ventricular volume; d/s: Diastole/systole. Here, *n* = 8.

**Table 2:** List of antibodies used in this study. All antibodies used in this study are listed with the corresponding distributor, catalog number, and dilution used for western blot.

Antibody	Manufacturer	Dilution
Phospho-p38	Cell Signaling Technology (9215)	1:1000
p38	Santa Cruz (sc-81621)	1:250
p53	Santa Cruz (sc-126)	1:250
JNK	Cell Signaling Technology (9252)	1:1000
BAX	Cell Signaling Technology (2772)	1:1000
